# Loss of RhoA in microglia disables glycolytic adaptation and impairs spinal cord injury recovery through Arhgap25/HIF-1α pathway

**DOI:** 10.1038/s41419-025-07947-9

**Published:** 2025-08-22

**Authors:** Jiale Cai, Xinya Zheng, Xiongbo Luo, Wenli Cui, Xinrui Ma, Shuyi Xu, Lanya Fu, Jiaqi Zhang, Yizhou Xu, Yunlun Li, Ye He, Xianghai Wang, Jiasong Guo

**Affiliations:** 1 Department of Histology and Embryology, Guangdong Provincial Key Laboratory of Construction and Detection in Tissue Engineering, National Demonstration Center for Experimental Education, School of Basic Medical Sciences, Department of Neurosurgery, Institute of Brain Diseases, Nanfang Hospital, Southern Medical University, Guangzhou, Guangdong Province China; 2 Key Laboratory of Mental Health of the Ministry of Education, Guangdong-Hong Kong-Macao Greater Bay Area Center for Brain Science and Brain-Inspired Intelligence, Guangdong Province Key Laboratory of Psychiatric Disorders, Guangzhou, Guangdong Province China; 3 Department of Spine Orthopedics, Zhujiang Hospital, Southern Medical University, Guangzhou, Guangdong Province China

**Keywords:** Spinal cord diseases, Microglia

## Abstract

RhoA, a small GTPase, plays a pivotal role in various diseases, including spinal cord injury (SCI). Although RhoA inhibition has been traditionally viewed as beneficial for SCI repair, recent clinical trials of RhoA inhibitors in SCI have failed to show significant therapeutic efficacy, suggesting functional heterogeneity across different cell types. The role of RhoA in microglia, the key immune cells involve in SCI, remains poorly understood. Using microglial RhoA conditional knockout mice, this study demonstrated that RhoA deficiency in microglia attenuates the morphological and functional repair of the SCI mice, and impairs the microglial biofunctions of proliferation, phagocytosis, and migration. Single-cell RNA sequencing, bulk RNA sequencing, and metabolomics revealed that RhoA deficiency can attenuate the microglial glycolytic enzyme expression, ATP production, ECAR and OCR levels through the Arhgap25/HIF-1α pathway. Overall, this is the first study to demonstrate that microglial RhoA is essential for SCI repair, the Arhgap25/HIF-1α pathway mediated glucose metabolism might enlighten a novel insight to enrich the understanding on the complex roles of RhoA and microglia in SCI repair. Moreover, this study highlights the importance of considering cell-specific roles of RhoA in SCI repair and provides a foundation for developing targeted therapies aimed at microglial metabolic reprogramming.

**Figure:**
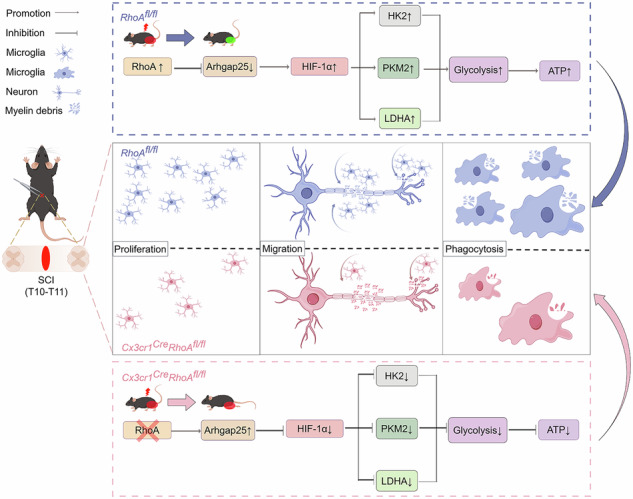
Schematic representation of the proposed mechanism by which microglial RhoA regulates glycolytic adaptation and spinal cord repair. (Created by Figdraw.com with permission of # wgq=r7c74c).

Schematic representation of the proposed mechanism by which microglial RhoA regulates glycolytic adaptation and spinal cord repair. (Created by Figdraw.com with permission of # wgq=r7c74c).

## Introduction

RhoA, a member of the small GTPase family, has garnered significant attention due to its marked upregulation or activation in various pathological conditions, including spinal cord injury (SCI) [1, 2] and cancer [3]. Historically, RhoA has been perceived as a detrimental molecule, with studies demonstrating that inhibiting its expression or activation can suppress tumor progression [4] and promote tissue repair [5, 6]. Such therapeutic benefits have been observed in conditions like erectile dysfunction [7], diabetic nephropathy [8], and atherosclerosis [9]. In the nervous system, substantial evidences indicate that suppressing RhoA can enhance neuronal survival and promote axonal regeneration [10, 11], making RhoA inhibitors promising candidates for treating SCI. However, recent multicenter, randomized, double-blind clinical trials have yielded unexpected results [12, 13]. RhoA inhibitors failed to achieve the anticipated therapeutic benefits for SCI, leading to the premature termination of the study based on interim futility analyses. This discrepancy between preclinical success and clinical failure raises critical questions. Why have interventions targeting RhoA not translated into clinical efficacy?

We hypothesize that the functional heterogeneity of RhoA across different cell types may underlie this disparity. For instance, neuron-specific RhoA knockout promotes SCI repair, while astrocyte-specific RhoA knockout appears to hinder recovery [14]. Similarly, our recent studies have demonstrated that RhoA conditional knockout in Schwann cells and macrophages exert opposing effects on peripheral nerve regeneration [15, 16]. Importantly, microglia play a central role in SCI recovery, and our previous study revealed a significant and sustained upregulation of RhoA in microglia post-SCI, persisting longer than in neurons or astrocytes [2]. This observation underscores the need to elucidate the specific role of RhoA in microglial function during SCI repair.

Microglia, as the resident immune cells of the central nervous system (CNS), are pivotal in mediating injury repair through processes such as proliferation, phagocytosis, and migration [17–19]. These functions are crucial for SCI recovery, making microglial modulation a key therapeutic strategy. Previous studies have highlighted RhoA’s diverse roles in microglia, including its regulation of glutamate release in Alzheimer’s disease [20], where RhoA deletion exacerbates disease progression, and ATP release [21], which is promoted by increased RhoA activity. Additionally, the RhoA/ROCK pathway has been implicated in the M1/M2 polarization of microglia and macrophages [22]. Beyond cytoskeletal dynamics, RhoA likely influences multiple biological processes through its involvement in diverse signaling pathways [23]. Does the sustained high expression of RhoA in microglia enhance their reparative functions, or does it facilitate SCI recovery by modulating metabolic pathways? Current researches have yet to provide definitive answers to these questions, making this an area of critical importance for understanding SCI repair and guiding therapeutic development.

In this study, we demonstrated the essential role of RhoA in microglia for regulating key functions such as proliferation, phagocytosis, and migration. Present findings revealed that RhoA modulates the glycolytic process in microglia via the Arhgap25/HIF-1α pathway, which offer a deeper understanding of its importance in SCI repair and indicate that targeted modulation of RhoA in microglia may therefore represent a promising strategy for improving clinical outcomes in SCI treatment.

## Materials and methods

### Animal procedures

All procedures were performed in accordance with guidelines set in place by the Southern Medical University Animal Care and Use Committee. C57BL/6 adult female mice (aged 8–12 weeks, weighing 20–25 g) and neonatal mice (days 2–3) were provided by the Animal Center of Southern Medical University. RhoA^flox/flox^ and Cx3cr1^Cre^ mice (provided by Cyagen, Suzhou, China) were intercrossed to obtain RhoA cKO mice with genotype of RhoA^flox/flox^; Cx3cr1^Cre^, as previously described [16]. The littermates with genotype of RhoA^flox/flox^ were utilized as negative controls for cKO mice.

### Spinal cord injury model

Mice spinal cord crush injury was performed at thoracic level 10-11 (T10-T11) similar to that described before [24]. Briefly, a midline incision was made over the thoracic vertebrae, followed by a T10-11 laminectomy. Tips of forceps were carefully inserted on either side of the cord to include the full width of the cord and then to gently scrape them across the bone on the ventral side to not spare any tissue ventrally or laterally. The spinal cord was fully crushed for 3 s with 0.1 mm forceps. The muscles were sutured, and the skin was closed with wound clips. Mice were placed on a warming pad after surgery until fully awake. Their bladders were manually expressed one times per day for the duration of the experiments.

### Behavioral assessments

The Basso Mouse Scale (BMS) was used to assess locomotor function at 1, 3, 7, 14, and 28-days post-injury (dpi) as previously reported. The BMS scale ranges from 0 (paralysis) to 9 (normal locomotion), scored during a 2–3 min open-field test by three blinded observers [25].

At 28 dpi, mice were trained to swim across a water-filled tank, with performance scored on hindlimb movement, coordination, tail position, paw placement, and balance [26]. The final score was the average of two trials, evaluated by two blinded investigators.

Four hours later, footprint analysis was performed to assess hindlimb function, with forelimbs and hindlimbs inked in black and red, respectively [27]. Mice ran along a paper-lined runway, and parameters like stride length, plantar stepping, and coordination were qualitatively analyzed.

### Histomorphometry of the gastrocnemius muscle

The harvested gastrocnemius muscles were weighted for calculating the wet weight [27]. Then mid-belly of the muscle was transversally sectioned and executed routine hematoxylin-eosin (HE) staining to visualize the myofibers. The area of myofibers was measured by using ImageJ software 6.0.

### Primary microglia culture

Primary microglia were isolated and cultured as previously described [28, 29]. In brief, a mixed glial cell culture was prepared from neonatal C57BL/6J mice and maintained for 10–21 d in DMEM (Thermo Fisher Scientific, MA, USA, C11995500BT) containing 10 ml of 10% FBS (Biochannel, Nanjing, China, BC-SE-FBS07) and 1% antibiotic-antimycotic (Thermo Fisher Scientific, MA, USA, 15240062) in a T75 flask. Microglia were collected by gentle shaking as the floating cells over the mixed glial cell culture. Then, primary microglia were transferred to a twelve-well or six-well plate for experiments.

### Myelin debris preparation

Myelin debris was prepared from the brains and spinal cords of mice as described previously [15]. Briefly, brain and spinal cord tissues were harvested from 8–10-week-old wild-type mice following euthanasia by cervical dislocation. The tissues were disrupted into fine particles via sonication, and the resulting debris was washed three times with Milli-Q water by centrifugation at 14,462 × *g* for 15 min at 4 °C. Finally, the myelin debris was resuspended in Hank’s balanced salt solution (HBSS, Thermo Fisher Scientific, MA, USA, C14175500BT) at a concentration of 100 mg/mL and stored at −80 °C for later use.

### Phagocytic capability assay

Microglial phagocytosis of myelin debris was assessed by seeding cells at 1 × 10⁵ cells/well in 12-well plates. After 24 h, 1 mg/mL myelin debris was added for 0.5, 1, or 2 h. After three washes with HBSS, protein content was measured, and cells were fixed with 4% PFA and stained with IBA-1 to visualize microglia morphology. Myelin debris uptake was co-stained with MBP for visualization.

### Migration assay

Microglial migration was assessed using a Transwell assay [15]. Cells were seeded in the upper chamber, with the lower chamber containing 600 µL DMEM, 10% FBS, and 1 mg/mL myelin debris. After 48 h, cells were fixed with 4% PFA, non-migrated cells on the upper membrane surface were removed, and migrated cells on the lower surface were stained with 0.1% crystal violet for 30 min. Images from five random fields were captured and quantified.

### Lipofectamine transfection for Arhgap25 knock down

Primary microglia were cultured to 60% confluence in six-well plates. Fifty nM siRNA targeting Arhgap25 or a negative control was transfected using 5 μL of Lipofectamine RNAi (D-Nano Therapeutics, Beijing, China), following the manufacturer’s protocol. The siRNA oligonucleotides were synthesized by Beijing D-Nano Therapeutics Co., Ltd. Sequences are provided in Supplementary Materials Table S1.

### Measurements of glucose and ATP

Primary microglia cells or culture medium were collected, and glucose and ATP were measured using glucose quantification colorimetric assay (Nanjing Jiancheng Bioengineering Institute, Nanjing, China, A154-1-1) and ATP bioluminescent assay kit (Beyotime Biotechnology, Shanghai, China, S0026). The contents of glucose and ATP were normalized by the total protein.

### ECAR and OCR measurement

After 24 h of treatment, the culture medium was removed, and ECAR (Elabscience, Wuhan, China, E-BC-F069) and OCR (Elabscience, Wuhan, China, E-BC-F068) were measured using commercial kits according to the manufacturer’s instructions. Fluorescence was recorded using a plate reader at 37 °C in dynamic mode. For ECAR, readings were taken every 2.5 min for 120 min (excitation: 490 nm; emission: 535 nm), and for OCR, every 2 min for 90 min (excitation: 405 nm; emission: 675 nm). Slopes of fluorescence curves were used to quantify ECAR and OCR.

### Rac1 activation assay

Primary microglia were transfected with Arhgap25 siRNA as described in 2.9 and then collected to detect the Rac1 activation using the Active Rac1 Detection Kit (NewEast Biosciences, China, 80501) following the manufacturer’s protocol.

### RNA sequencing

Primary microglia from the flox and cKO groups were sequenced using Solexa high-throughput sequencing (Biotree, Shanghai, China). RNA was extracted with Trizol reagent, and sequencing was followed by data analysis, including Venn analysis, GO, and KEGG enrichment analysis.

### Targeted metabolomics

Primary microglia were seeded at 5 × 10⁶ cells/dish. After 24 h, cells were washed with HBSS, collected, and subjected to metabolite extraction. The supernatant was analyzed by LC-MS using an ExionLC system and an AB Sciex Triple Quadrupole 6500 mass spectrometer in MRM mode. Data visualization was done using the BioDeep cloud platform.

### Single-cell RNA sequencing and data analyses

Raw data from the 10× Genomics platform were processed using Cell Ranger and imported into R for analysis with Seurat. Cells were filtered, and batch effects corrected using Harmony. PCA was performed with the top 2000 variable genes, and cell types were annotated using the SingleR package. Differentially expressed genes were identified using Seurat’s FindAllMarkers function. GO, KEGG, and GSEA were performed with the clusterProfiler package, considering terms with *p* value < 0.05 and *q*-value < 0.05.

### Public microglia sequencing data analysis

Microglia sequencing data were downloaded from the GEO database [24, 30–32]. Detailed information is provided in Supplementary Materials Table S2.

### Immunostaining

After perfusing mice with 0.1 mol/L PBS and 4% PFA, spinal cords were fixed in 4% PFA for 24 h and then immersed in 30% sucrose until fully submerged. A 1 cm spinal cord segment was cut into 10-μm thick sections using a freezing microtome. For staining, sections were fixed for 30 min, antigen-retrieved with Sodium Citrate Solution at 90 °C for 30 min, and blocked in 5% BSA and 0.3% Triton X-100 for 1 h. Sections were incubated overnight with primary antibodies at 4 °C, then washed with PBS and incubated with secondary antibodies for 1 h at room temperature. Primary antibody details are in Supplementary Materials Table S3. Sections were stained with DAPI to visualize the nucleus, and images were acquired with light and confocal microscopes, then analyzed using ImageJ and Photoshop.

### Western blotting

Microglia were lysed in ice-cold RIPA Buffer (FDbio, Hangzhou, China, FD009) with 100 mM PMSF, incubated at 4 °C for 30 min, and extracted with 5× loading buffer. Proteins were boiled at 100 °C for 10 min and separated using SDS-PAGE before being transferred to nitrocellulose membranes (Merck Millipore, Darmstadt, Germany, ISEQ00010). After blocking with 5% skim milk for 1 h, membranes were incubated overnight with primary antibodies at 4 °C, washed, and incubated with HRP-conjugated secondary antibodies for 2 h. The western blot was detected using the ECL detection kit (Biosharp, Anhui China, BL523B), and images were analyzed with ImageJ software.

### Statistical analysis

All statistical analyses were performed in Prism Version 8.0.2 (GraphPad). Data were expressed as mean ± SD from at least three independent experiments. statistical significance was assessed by the Student’s *t* test for comparison between two groups or ANOVA with post hoc tests by Bonferroni correction for comparisons among three or more groups; *p* < 0.05 was considered statistically significant.

## Results

### RhoA expression is upregulated in microglia following spinal cord injury

**Fig. 1:**
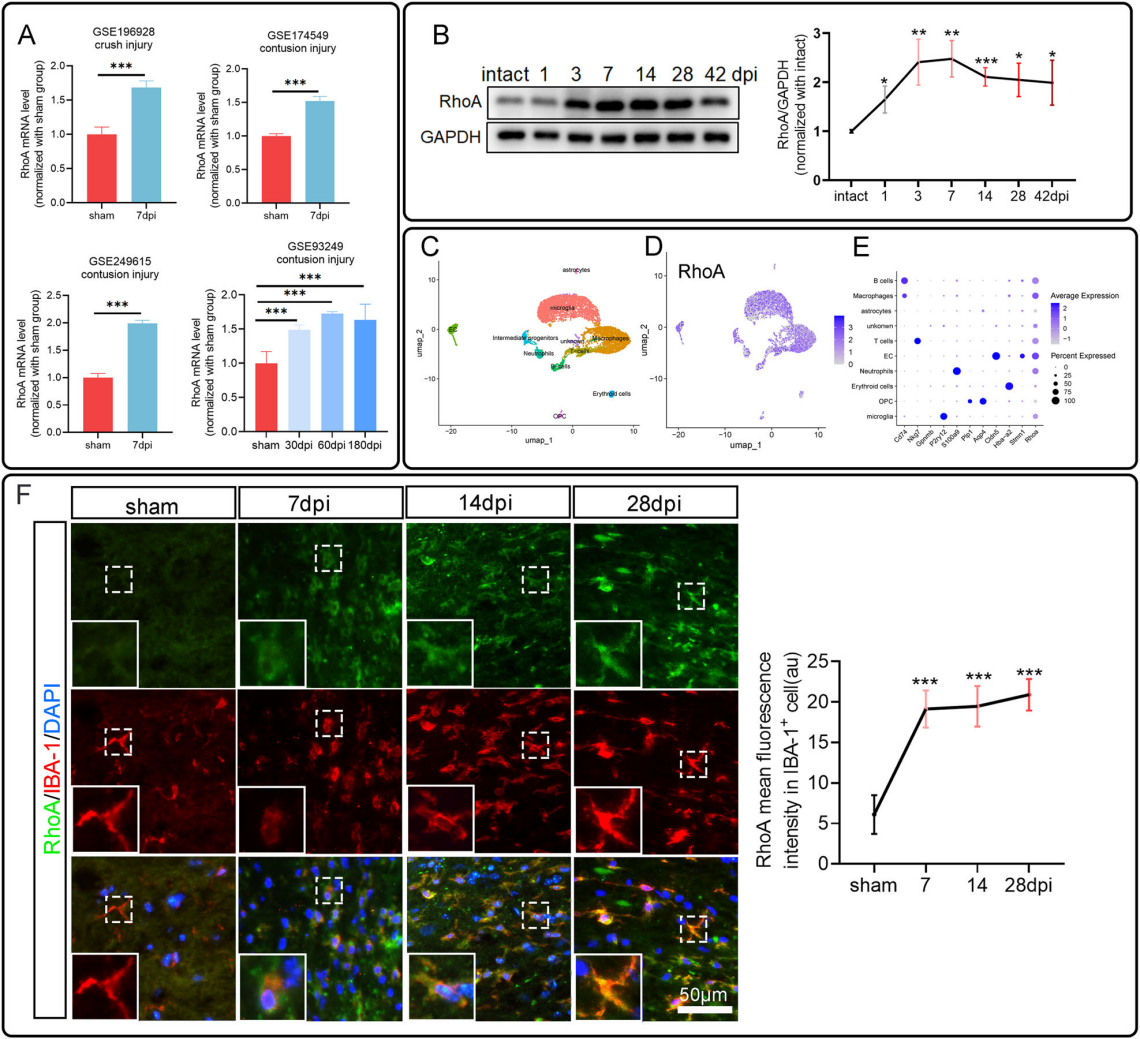
RhoA expression in microglia was up-regulated by spinal cord injury. **A** Changes of RhoA mRNA expression in injured spinal cord obtained from different GEO databases. **B** Temporal changes of RhoA protein level in spinal cord following SCI (*n* = 5). **C** Major cell type classification in the GSE196928 dataset. **D** Distribution map of RhoA expression. **E** RhoA expression levels across different cell types. **F** Immunostaining shows the RhoA expression in microglia (IBA-1^+^) at different stages after SCI (*n* = 3). Data are presented as mean ± SD. Statistical significance: ∗*p* < 0.05, ∗∗*p* < 0.005, ∗∗∗*p* < 0.001 (vs. sham group).

In the past, studies mainly focused on the activity of RhoA, but recently the total amount of RhoA changes has been found in several diseases. By analyzing the GEO databases, it was observed that RhoA expression is elevated following SCI and is significantly increased across multiple datasets (Fig. 1A). Western blotting assay on the injured spinal cord tissues at 1, 3, 7, 14, 28, and 42 days post injury (dpi) revealed an increase in RhoA protein level (Fig. 1B), which is consistent with previous reports, and indicated that RhoA is a key regulatory factor in SCI. By sorting out the GSE196928 dataset, RhoA was found that extensively expressing in microglia after SCI (Fig. 1C–E). Immuno-staining illustrated that RhoA expression in microglia was increased after SCI (Fig. 1F). These findings suggest that the change of RhoA expression in microglia might be involved in SCI repair.

### Microglial RhoA deficiency delays the morphological and functional recovery in the spinal cord injury mice

**Fig. 2:**
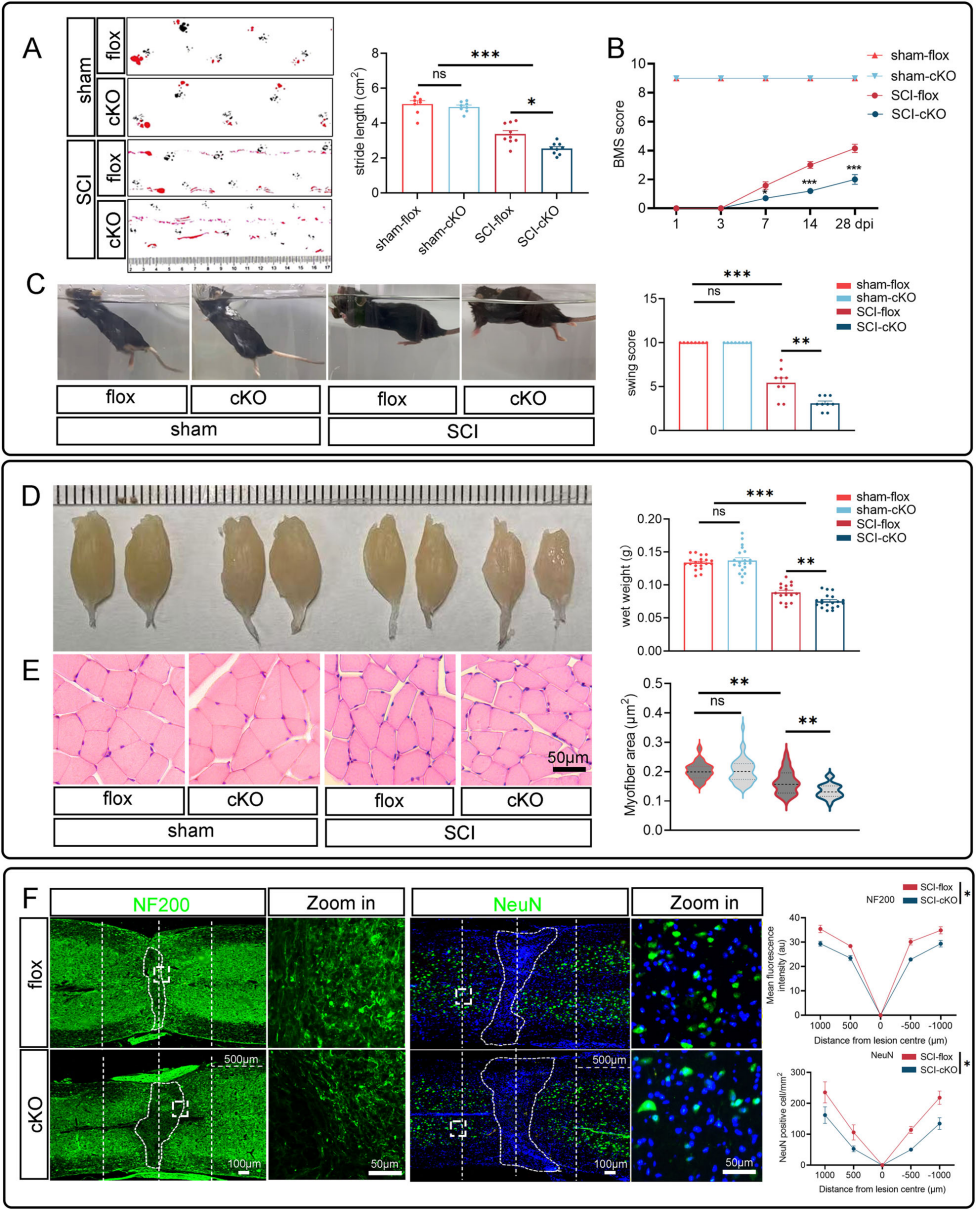
Microglial RhoA deficiency delays the spinal cord injury recovery. **A** Representative foot print images of mice at 28 dpi. **B** BMS scores at different stages after SCI. **C** Swimming test was used to evaluate locomotor function in mice at 28 dpi. **D** Gastrocnemius muscle weight at 28 dpi. **E** H&E staining of the gastrocnemius muscle at 28 dpi. **F** NF200 Mean fluorescence intensity and the number of NeuN-positive cells at various distances from the SCI lesion center of the spinal cords present 1000 μm proximal (*n* = 3). Data are presented as mean ± SD; each dot represents an individual mouse. Statistical significance: n.s. nonsignificant; ∗*p* < 0.05; ∗∗*p* < 0.01; ∗∗∗*p* < 0.001.

To investigate the role of microglial RhoA in SCI, we created a microglial RhoA conditional knockout (cKO) mouse model. Western blots and immunostaining confirmed successful RhoA ablation in microglia in the cKO mice (Supplementary Fig. S1). The cKO and RhoA-flox (flox, control) mice underwent SCI or sham surgery. At 28 dpi, in the SCI group, the cKO mice showed significantly worse BMS scores, swimming test results, and gait analysis scores compared to the flox group, while there were no significant differences between the two groups in the sham-treated mice (Fig. 2A–C). These results suggest that RhoA deficiency impairs functional recovery after SCI.

The recovery of motor function after SCI depends heavily on gastrocnemius muscle atrophy. At 28 dpi, the gastrocnemius muscle weight was significantly lower in the cKO group compared to the flox group. HE staining further revealed a notable reduction in myofiber area in the cKO group (Fig. 2D, E).

Immunostaining for NF200 (axon marker) and NeuN (neuron marker) was used to assess spinal cord morphology. Results showed a significant decrease in NF200 immuno-intensity and the number of neurons in the para-epicenter of the injured spinal cord in the cKO group (Fig. 2F). These findings suggest that microglial-specific RhoA knockout impairs both morphological and functional repair after SCI.

### RhoA deficiency alleviates the microglial capabilities of proliferation, phagocytosis, and migration

**Fig. 3:**
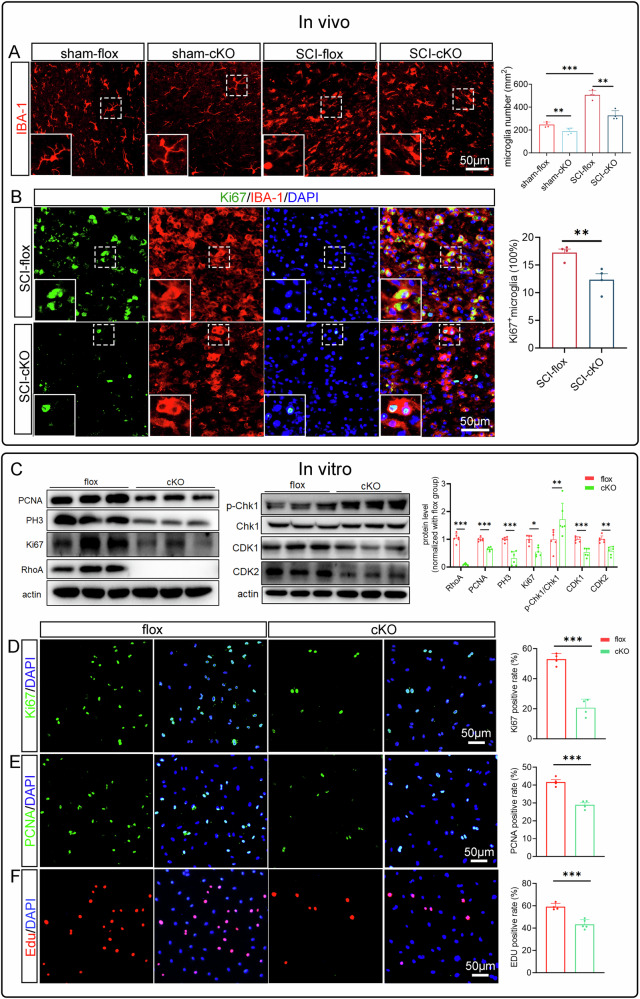
RhoA knockout inhibits the microglial proliferation. **A** Immunostainings show the number of Microglia (Iba-1^+^) cells in spinal cord tissue at 28 dpi. **B** Immunostainings show Ki67^+^ (green) and IBA-1^+^ (red) microglial cells in the SCI at 7 dpi. **C** Western blotting shows the related protein levels of proliferating and cell cycle in the primary cultured microglia. Immunostainings show the Ki67^+^ cells (**D**), PCNA^+^ cells (**E**) and Edu^+^ cells (**F**) in primary cultured microglia. Data are presented as mean ± SD; each dot represents an individual mouse. Statistical significance: ∗*p* < 0.05; ∗∗*p* < 0.01; ∗∗∗*p* < 0.001.

Using IBA-1 immunostaining to label microglia, we found that the number of microglia in both injured and uninjured spinal cord sections at 28 dpi was significantly lower in the cKO group compared to the flox group (Fig. 3A). This suggests that RhoA may influence microglial death or proliferation. To explore this, we examined markers for ferroptosis (SLC7A11, GPX4), cuproptosis (FDX1), autophagy (LC3B), and apoptosis (Bcl2/Bax) (Supplementary Fig. S2). The results showed that RhoA knockout inhibited apoptosis and promoted autophagy, but had no significant effect on ferroptosis or copper-induced cell death. These findings suggest that the reduced microglial number in the cKO group is primarily due to impaired proliferation rather than increased cell death.

To confirm this, we performed Ki67/IBA-1 double immunostaining at 7 dpi, when microglial proliferation peaks. The data showed a significantly lower ratio of proliferating cells (Ki67+/IBA-1+) in the cKO group compared to the flox group (Fig. 3B). In primary microglial cultures, RhoA knockout significantly reduced the expression of proliferative markers (PCNA, PH3, Ki67, CDK1, CDK2) and increased cell cycle arrest markers (p-Chk1/Chk1) (Fig. 3C). Immunostaining also showed a significant decrease in Ki67+, PCNA+, and EdU+ microglia in cKO cultures compared to flox cultures (Fig. 3D–F).

**Fig. 4:**
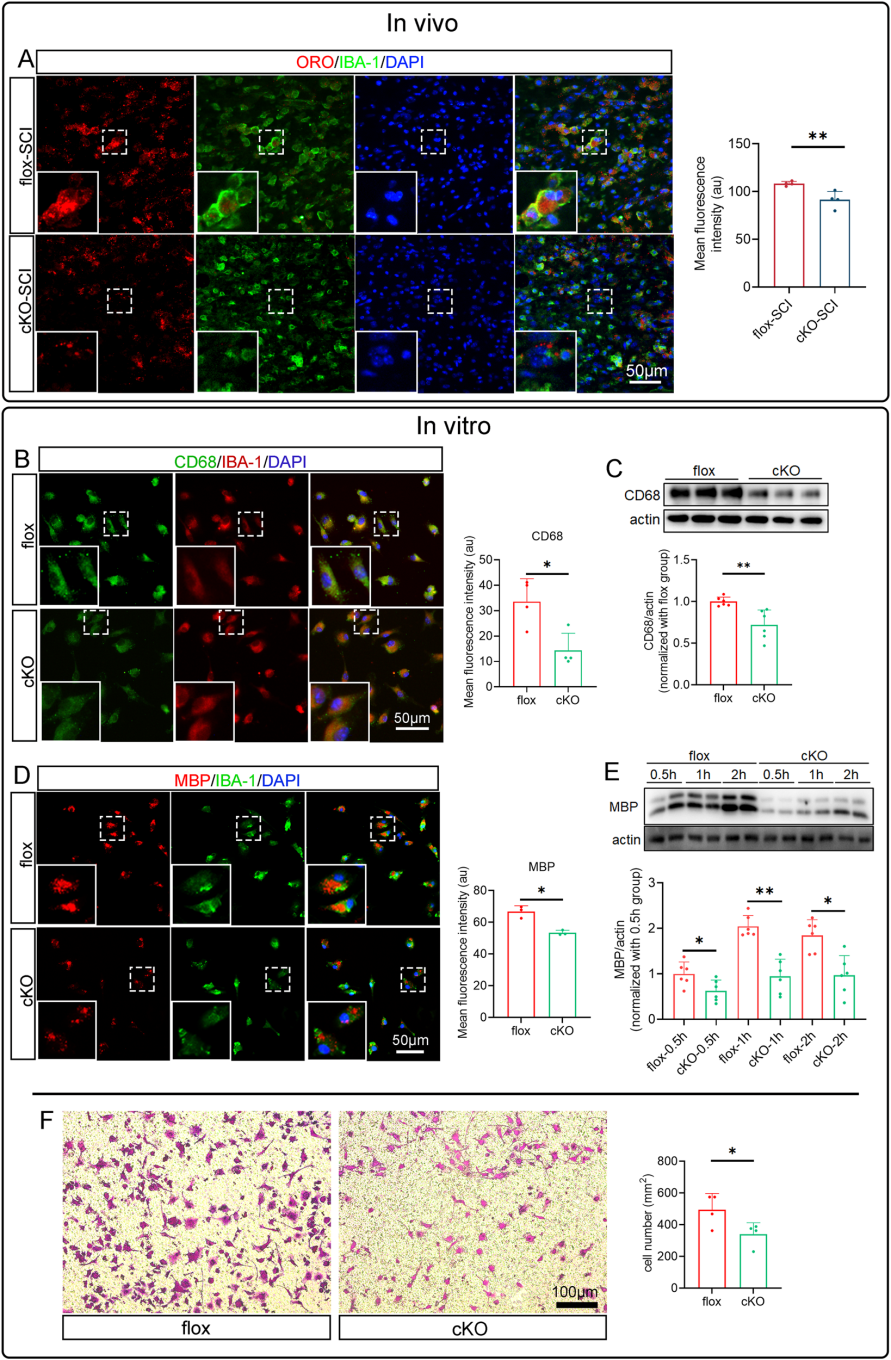
RhoA knockout attenuates the microglial phagocytosis and migration. **A** Immunostainings show IBA-1^+^ and ORO^+^ on the cross sections of the 7 dpi spinal cord tissue. **B**, **C** Immunostaining and Western blotting show the CD68 expression in the cultured microglia (IBA-1^+^). **D**, **E** Immunostaining and Western blotting demonstrate microglia engulfed myelin debris (MBP^+^) in the myelin debris treated microglial cultures. **F** Representative images of Transwell assay. Data are presented as mean ± SD; each dot represents an individual mouse. Statistical significance: ∗*p* < 0.05; ∗∗*p* < 0.01.

Microglial phagocytosis is critical for clearing debris following SCI, so we assessed this function in vivo and in vitro. IBA-1 and ORO staining revealed fewer ORO+ lipid droplets in cKO microglia in the injured spinal cord at 7 dpi (Fig. 4A). In vitro, the phagocytic capacity was evaluated by measuring myelin fragment uptake over time. CD68, a phagocytosis marker, showed significantly lower fluorescence intensity protein level in the cKO group (Fig. 4B, C). Additionally, MBP+ myelin debris content was reduced in the cKO group compared to the flox group (Fig. 4D, E). These findings indicate that RhoA is essential for maintaining microglial phagocytic function.

Finally, since migration is important for microglial roles in SCI repair, we performed a Transwell assay and found that RhoA knockout significantly impaired microglial migration (Fig. 4F). This suggests that RhoA is crucial for microglial migration.

### Multi-omics analysis reveals RhoA regulation of the glycolysis and oxidative phosphorylation in microglia

**Fig. 5:**
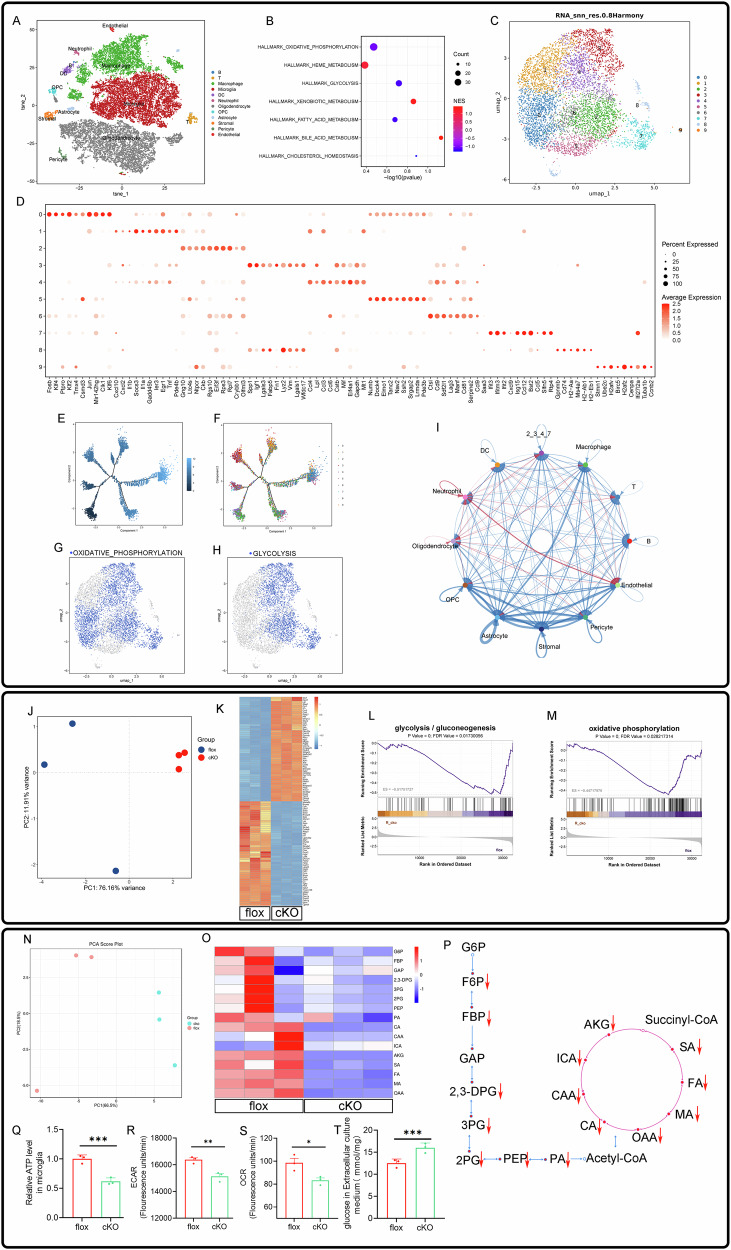
Multi-omics insights into RhoA-mediated regulation of glycolysis and oxidative phosphorylation in microglia. **A** t-SNE and unsupervised clustering of all cells based on biomarkers. **B** GSEA analysis highlighting differences in microglial subsets compared to other cell types. **C** High-resolution reclustering of microglia into ten distinct clusters. **D** Expression of key markers identified in the reclustered microglia. **E** Pseudotime trajectory analysis of microglial cells. **F** Visualization of ten microglial subclusters, each represented by a distinct color. Microglial subsets with significant differences in oxidative phosphorylation pathways (**G** subcluster 0, 2, 3, 4, 7) and glycolysis pathways (**H** subcluster 2, 3, 4, 7) identified through GSEA analysis. **I** CellChat analysis showing interactions between glycolysis-differentiated microglia (subcluster 2, 3, 4, 7) and other cell types. **J** PCA of bulk mRNA transcriptomics in primary microglial cells. **K** Heatmap illustrating the results of mRNA sequencing analysis. GSEA analysis of glycolysis-related (**L**) and oxidative phosphorylation-related (**M**) differences between flox and cKO groups. **N** PCA of metabolomic profiling data. **O**, **P** Comparative analysis of glycolysis and TCA cycle metabolic changes between cKO and flox groups. RhoA deletion decreases ATP production (**Q**), accompanied by reduced ECAR (**R**), OCR (**S**), and glucose consumption in the culture medium (**T**). Data are presented as mean ± SD; each dot represents an individual mouse. Statistical significance: ***p < 0.001.

To investigate how microglial RhoA knockout affects SCI repair, we performed single-cell RNA sequencing (scRNA-Seq) at 7 dpi. After basic annotation and dimensionality reduction using t-SNE, we identified 12 cell clusters, including microglia, macrophages, astrocytes, and oligodendrocytes (Fig. 5A and Supplementary Fig. S3A). Given the importance of metabolic reprogramming in microglial activity, we focused on metabolism. GSEA analysis of microglial clusters revealed that oxidative phosphorylation and glycolysis were significantly downregulated in the cKO group compared to the flox group (Fig. 5B). Further analysis of microglia identified 10 subclusters and their markers (Fig. 5C, D). Pseudotime analysis showed the distribution of subclusters along the pseudotime trajectory (Fig. 5E, F). Supplementary Fig. S4 displays the top 20 GSEA results for each subcluster. Notably, glycolysis was significantly altered in subclusters 2, 3, 4, and 7, while oxidative phosphorylation was affected in subclusters 0, 2, 3, 4, and 7. The UMAP plot highlighted clusters linked to these pathways (Fig. 5G, H). CellChat interaction analysis suggested that microglial subclusters with altered metabolism interact with astrocytes, endothelial cells, neurons, and oligodendrocytes (Fig. 5I and Supplementary Fig. S5). Overall, scRNA-Seq data suggest that RhoA knockout modulates glycolysis and oxidative phosphorylation in microglia, influencing their function post-SCI.

To explore RhoA’s role further, we isolated primary microglia and performed bulk mRNA sequencing. PCA analysis showed clear separation between the flox and cKO groups (Fig. 5J), indicating gene expression changes due to RhoA knockout. The heatmap highlighted significant gene expression differences (Fig. 5K). GSEA analysis confirmed that glycolysis and oxidative phosphorylation were downregulated in the cKO group (Fig. 5L, M), consistent with scRNA-Seq results. Targeted metabolomics revealed metabolic changes between the two groups, with PCA analysis showing clear separation (Fig. 5N). Alterations in glycolysis-related and TCA cycle metabolites are shown in Fig. 5O, with pathway mapping in Fig. 5P. ATP, ECAR, and OCR measurements revealed that ATP levels, as well as ECAR and OCR values, were reduced in cKO microglia (Fig. 5Q–S). In addition, glucose consumption was higher in the flox group, as indicated by the decreased glucose concentration in the culture medium (Fig. 5T). Together, scRNA-Seq, mRNA sequencing, and metabolomics data suggest that RhoA knockout in microglia alters glycolysis and oxidative phosphorylation, impacting their biological function.

### Arhgap25/HIF-1α pathway is involved in RhoA regulating microglial biofunctions

**Fig. 6:**
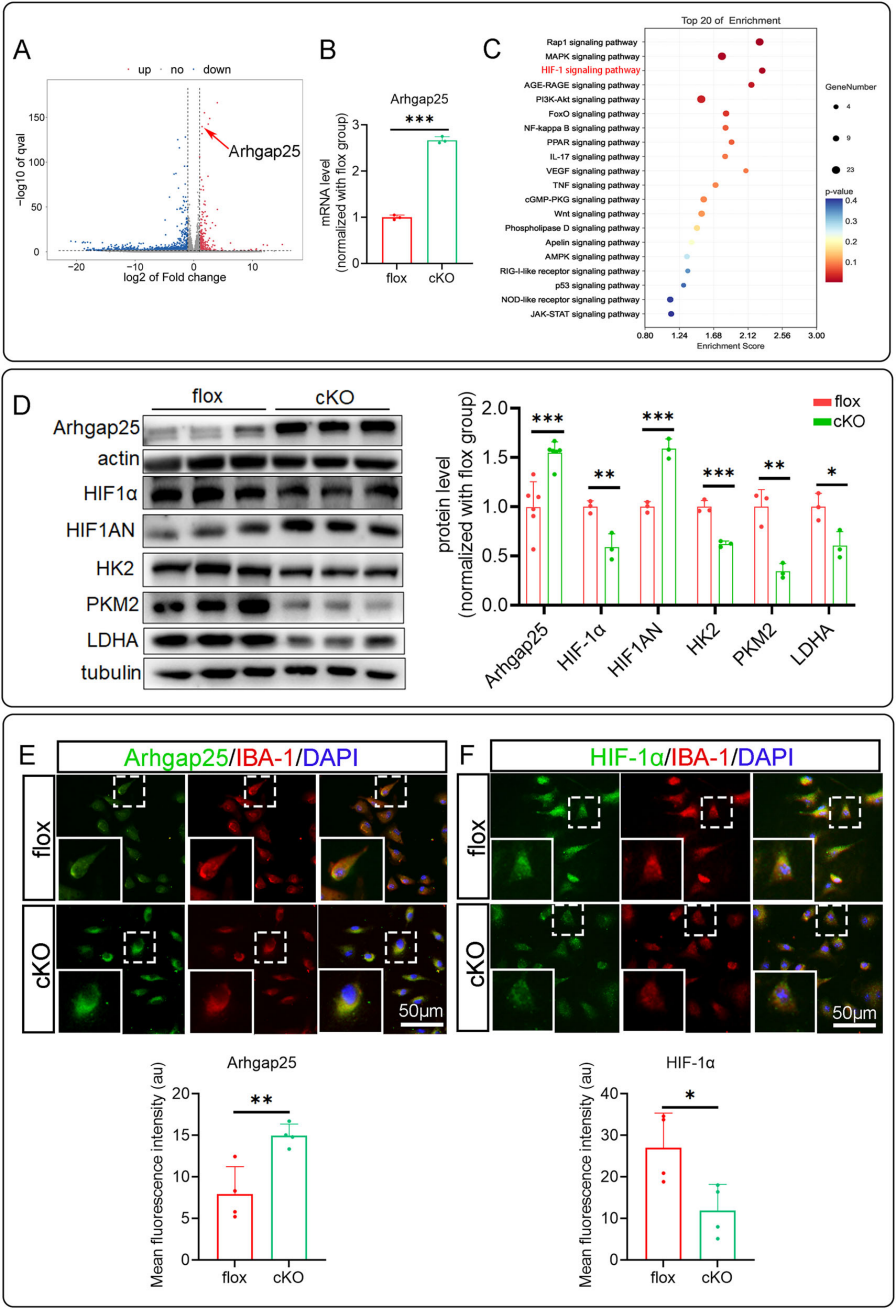
RhoA regulates the microglial biofunctions by regulating the HIF-1 pathway through Arhgap25. **A** Highlight Arhgap25 in volcano diagram of mRNA-sequence. **B** *Arhgap25* mRNA level in primary microglia in mRNA-sequence. **C** KEGG enrichment analysis of significant changes gene in mRNA-sequence. **D** Western blots show the Arhgap25, HIF-1α, HIF1AN, HK2, PKM2 and LDHA protein expression levels in primary cultured microglia. **E**, **F** Immunostainings show Arhgap25 and HIF-1α expression in primary cultured microglia. Data are presented as mean ± SD; each dot represents an individual mouse. Statistical significance: ∗*p* < 0.05; ∗∗*p* < 0.01; ∗∗∗*p* < 0.001.

The aforementioned results suggested that RhoA regulates microglial biofunction through metabolic reprogramming, however which signaling pathway is involved in this regulation remains unclear. To figure out this issue, our primary cultured microglia mRNA transcriptomic analysis revealed significant differential expression of *Arhgap25*, as shown in the volcano plot (Fig. 6A, B). Additionally, KEGG pathway enrichment analysis indicated that RhoA knockout affects the HIF-1 pathway (Fig. 6C). Previous studies have demonstrated that Arhgap25 regulates the proliferation of human pancreatic cancer cells via HIF-1α [33], leading us to hypothesize that RhoA modulates glycolytic processes through Arhgap25/HIF-1α pathway. Therefore, main components of this pathway were detected by Western blotting. The results showed a significant increase in Arhgap25 expression in the cKO group, accompanied by a decrease in HIF-1α protein levels and a corresponding increase in HIF1AN protein levels (Fig. 6D). Furthermore, the expression of key glycolytic enzymes, HK2, PKM2 and LDHA, were reduced. Immunostaining confirmed that Arhgap25 expression was significantly higher, while HIF-1α expression was markedly lower in the cKO group (Fig. 6E, F).

### Arhgap25 knockdown reverses the effects of RhoA deficiency on the microglial biological functions

**Fig. 7:**
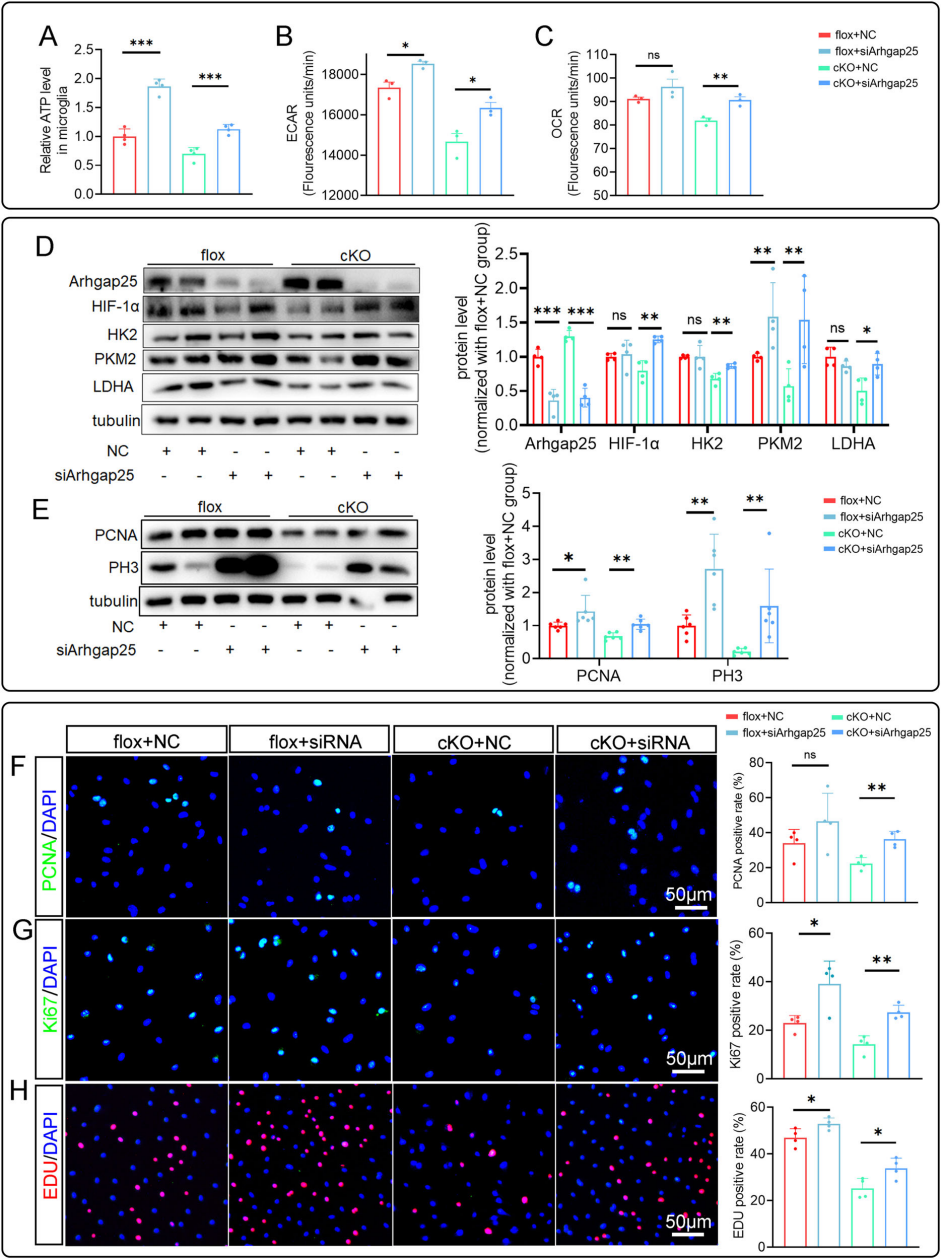
Arhgap25 knockdown reverses the regulatory effect of RhoA knockout on the biological function of microglia. ATP concentration (**A**), ECAR (**B**) and OCR (**C**) levels in primary cultured microglia after siArhgap25 transfection. **D** Western blotting shows the expression of Arhgap25, HIF-1α, HK2, PKM2 and LDHA after siArhgap25 transfection. **E** Western blotting shows the expression of PCNA and PH3 protein after siArhgap25 transfection. Immunostaining shows the PCNA^+^(**F**), Ki67^+^ (**G**) and Edu^+^ (**H**) in primary cultured microglia after transfection with siArhgap25. Data are presented as mean ± SD; each dot represents an individual mouse. Statistical significance: ∗*p* < 0.05; ∗∗*p* < 0.01; ∗∗∗*p* < 0.001.

To validate if the Arhgap25/HIF-1α pathway is involved in RhoA regulation of microglial functions, we developed three siRNAs targeting Arhgap25. Among them, siArhgap25-1 showed the best knockdown efficiency in primary microglia (Supplementary Fig. S6) and was used in further experiments. Arhgap25 knockdown enhanced cellular metabolism, as reflected by increased ATP production, ECAR, and OCR in the cKO group (Fig. 7A–C), suggesting it reversed the energy deficiency caused by RhoA knockout. Moreover, protein levels of HIF-1α, HK2, PKM2, and LDHA were significantly elevated in the cKO group after Arhgap25 knockdown (Fig. 7D). These results suggest that RhoA regulates microglial metabolism through the Arhgap25/HIF-1α pathway.

**Fig. 8:**
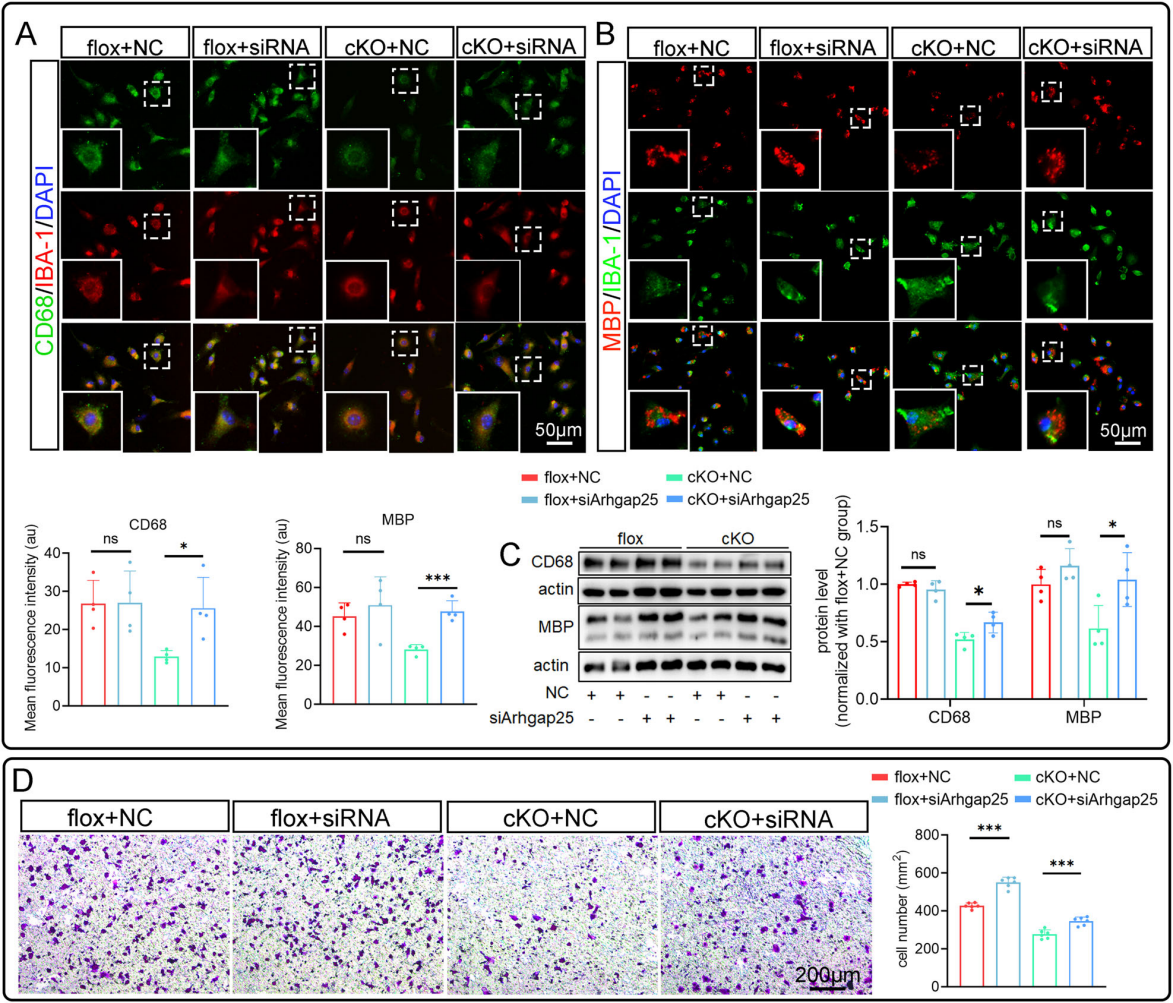
Arhgap25 knockdown reverses the effects of RhoA cKO on the phagocytosis and migration of microglia. **A** Immunostaining shows the CD68 expression in the cultured microglia (IBA-1^+^). **B** Immunostaining shows the myelin debris (MBP^+^) engulfed in the cultured microglia after the microglia were co-cultured with debris for 1 h. **C** Western blotting show the CD68 level in microglia (culture without debris) and MBP level in microglia (co-cultured with debris for 1 h). **D** Representative images of Transwell assay. Data are presented as mean ± SD; each dot represents an individual mouse. Statistical significance: ∗*p* < 0.05; ∗∗*p* < 0.01; ∗∗∗*p* < 0.001.

We then assessed the effects of siArhgap25 on microglial proliferation. SiArhgap25 reversed the proliferation inhibition in the cKO group, as indicated by increased PCNA and PH3 expression (Fig. 7E), and consistent results from PCNA, Ki67, and Edu staining (Fig. 7F–H). For phagocytic function, CD68 and MBP staining and Western blotting showed that siArhgap25 restored phagocytosis in cKO microglia (Fig. 8A–C). Additionally, Transwell migration assays revealed that siArhgap25 reversed the migration impairment in the cKO group (Fig. 8D). In summary, these findings indicate that RhoA regulates microglial functions-proliferation, phagocytosis, and migration-via the Arhgap25/HIF-1α pathway and metabolic reprogramming.

## Discussion

Research on SCI has spanned several decades, yielding numerous exciting breakthroughs. In particular, recent advancements in brain-spinal interface [34–36] and epidural electrical stimulation [37–39] have shown significant clinical efficacy. However, it is regrettable that despite extensive studies aimed at improving the microenvironment or the neuronal intrinsic regenerative capability to promote SCI repair, successful clinical applications remain rare [12, 13, 40]. This underscores the inherent complexity of SCI pathophysiology.

Following SCI, fragmented myelin sheaths, dead cells, and activated inflammatory factors release a variety of inhibitory molecules that impede neural regeneration [41–44]. Although previous approaches, such as antibodies or enzymes targeting chondroitin sulfate proteoglycans (CSPGs), have attempted to mitigate these inhibitors, they fail to comprehensively clear all inhibitory factors [45]. Subsequent researches identified RhoA signaling as a critical pathway mediating the effects of many inhibitory molecules in the SCI microenvironment [45]. Up to now, the role of RhoA as a negative regulator of axonal regeneration has been well established [14, 46, 47]. Our recent study also revealed that neuronal-specific RhoA deletion delays dendritic degeneration and promotes dendritic regeneration after the axonal injury [48]. All the existing findings lead to the hypothesis that inhibiting RhoA could promote SCI repair, yet RhoA-targeted inhibitors have failed to meet expectations in clinical trials, emphasizing the urgent need to further explore the role and mechanisms of RhoA in SCI [12, 13].

Following SCI, RhoA upregulation and activation are not confined to neurons; these also occur in astrocytes and microglia [2, 49, 50]. Recent studies with RhoA conditional knockout mice demonstrated that neuronal RhoA deficiency accelerates the SCI repair, while astrocyte RhoA depletion attenuates the SCI recovery [14]. Compared to neuron and astrocyte, microglia in the injured spinal cord exhibit the more significant and sustained upregulation of RhoA [24, 51]. Given the pivotal role of microglia in SCI, this raises an intriguing question: whether RhoA plays pivotal role in microglial biofunctions, and how do they influence SCI repair?

Herein, microglia-specific RhoA knockout mice were used to address above questions. Collected data revealed that RhoA deletion in microglia significantly impeded the structural and functional recoveries post-SCI. To our knowledge, this is the first study to reveal that microglial RhoA is essential for SCI repair. Even some studies mentioned the RhoA playing role in spinal cord injury is related to microglial inflammation [52], their manipulations are not cell-specific, so their results might be interrupted by RhoA’s roles in other cells but not only microglia.

Secondly, this study firstly reveals the role of RhoA in microglial capabilities of proliferation, migration and phagocytosis. Previous studies mainly focused on that RhoA regulates microglia producing inflammatory factors [53], nobody reports the proliferation, migration and phagocytosis in spinal cord injury. While, these biofunctions are critical for microglia to play role in Wallerian degeneration after SCI and produce favorable microenvironment for axonal regeneration [54–57].

Thirdly, this study provides a novel signaling pathway to explain the role of RhoA in microglia. Arhgap25/HIF-1α pathway mediated glucose metabolism might enlighten a novel insight to enrich our understanding on the complex role of RhoA. Although RhoA has long been regarded as an important switching molecule in regulating actin cytoskeleton reorganization [58], recent studies have also shown that RhoA can affect microtubule dynamics [59] or the expressions of certain transcription factors [60, 61] independently of actin dynamics. Herein, a series results of sequence analyses and validation experiments revealed that RhoA deletion in microglia alters the expression of the cytoskeleton-related molecules (mDia and ROCK2) (Supplementary Materials Fig. S7A, B). Which indicate cytoskeletal remodeling is also involved in the role of RhoA in microglia. Noticeably, we identified that RhoA depletion reduced glycolytic activity and ATP production through Arhgap25/HIF-1α signaling. Consequently, energy deficiency impairs microglial proliferation and migration, reducing the microglial population and diminishing phagocytic capacity. These impairments hinder the timely clearance of dead cells and debris in the SCI microenvironment, ultimately compromising structural and functional recoveries. Present data also illustrated that siArhgap25 resulted in a higher ratio of Rac1-GTP/ total Rac1 in the primary cultured microglia, which means suppression of Arhgap25 promotes Rac1 activation (Supplementary Materials Fig. S7C). This is consistent with the previous studies of that [62–64], Rac1-GTP has been shown to enhance cell proliferation, migration, and glycolytic activity. However, the exact role of Rac1 activation in RhoA modulating glucose metabolism in microglia remains unclear. Further investigation is needed to clarify the potential crosstalk between RhoA and Rac1 signaling in microglial function.

## Conclusion

In summary, this is the first study to reveal that the SCI resulted RhoA upregulation in microglia plays positive roles in SCI repair which may due to RhoA regulating microglial bio-functions of proliferation, migration and phagocytosis through the Arhgap25/HIF-1α pathway mediated modulating glycolysis. Even RhoA inhibition in neurons is widely regarded as a prospective strategy for promoting axonal regeneration after SCI [65], recent study indicated astrocytic RhoA specific knockout alleviates SCI repair [14] and the evidences of this study demonstrated microglial RhoA specific knockout is also not conducive to SCI repair, which suggests that RhoA inhibition for SCI repair has to consider the cell specific targeting. Moreover, this study hints that targeting Arhgap25/HIF-1α pathway to regulate microglial glucose metabolism might be another potential therapeutic strategy for SCI.

## Supplementary information

supplementary data

WB-RAW-DATA

## Data Availability

The data that supporting the findings of this study will be made available by the corresponding authors upon reasonable request. The RNA sequencing data generated in this study have been deposited in the Genome Sequence Archive (GSA) under accession number PRJCA040700, and will be made publicly available upon publication.
