# Supplementary material for: Loss of RhoA in microglia disables glycolytic adaptation and impairs spinal cord injury recovery through Arhgap25/HIF-1α pathway
Source: Cell Death Dis. 2025 Aug 22;16(1):636. doi: 10.1038/s41419-025-07947-9 (PMC12373832; doi:10.1038/s41419-025-07947-9)
Supplement: Supplementary file 1 — supplementary data [file 41419_2025_7947_MOESM1_ESM.pdf]

supplementary material for

Loss of RhoA in microglia disables glycolytic adaptation and impairs spinal cord injury recovery through Arhgap25/HIF-1α pathway

Jiale Cai [a, b†], Xinya Zheng [a†], Xiongbo Luo [a], Wenli Cui [a], Xinrui Ma [a, b], Shuyi Xu [a, b], Lanya Fu [a], Jiaqi Zhang [a], Yizhou Xu [a, c], Yunlun Li [a], Ye He [a], Xianghai Wang [a, b], Jiasong Guo [a, b, c*]

a. Department of Histology and Embryology, Guangdong Provincial Key Laboratory of Construction and Detection in Tissue Engineering, National Demonstration Center for Experimental Education, School of Basic Medical Sciences; Department of Neurosurgery, Institute of Brain Diseases, Nanfang Hospital; Southern Medical University, Guangzhou, Guangdong Province, China

b. Key Laboratory of Mental Health of the Ministry of Education, Guangdong-Hong Kong-Macao Greater Bay Area Center for Brain Science and Brain-Inspired Intelligence, Guangdong Province Key Laboratory of Psychiatric Disorders, Guangzhou, Guangdong Province, China

c. Department of Spine Orthopedics, Zhujiang Hospital, Southern Medical University, Guangzhou, Guangdong Province, China

† Jiale Cai and Xinya Zheng contributed equally to this work.

*Correspondence to

Professor Jiasong Guo, Southern Medical University, Guangzhou 510515, China; jiasongguo@smu.edu.cn.

This file includes:

Figures S1 to S7 and Table S1-S3

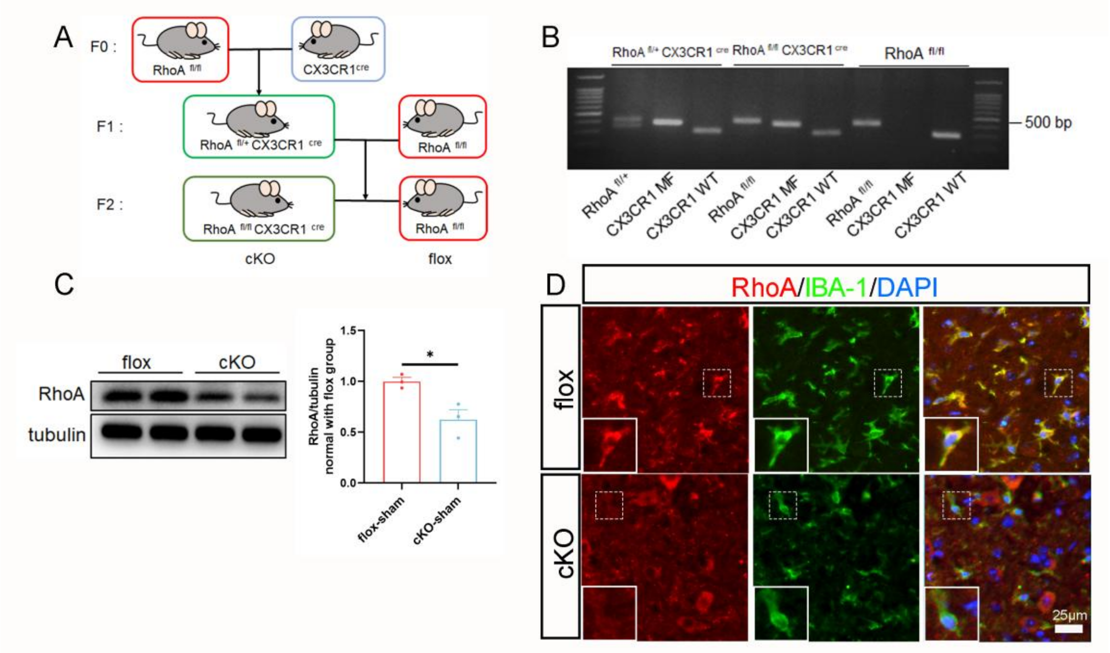

Figure S1. Identification of the microglial RhoA conditional knockout (cKO) mice. (A) The cKO (RhoA $^{flox/flox}$; CX3CR1^{cre}) mice were generated by crossing RhoA $^{flox/flox}$ mice to CX3CR1^{cre} driver lines. (B) Representative image of genotyping. (C, D) Western blotting and immunostaining show the RhoA expression in the spinal cord tissue of the RhoA $^{flox/flox}$ (Flox) mice and cKO mice. Data are presented as mean ± SD; each dot represents an individual mouse. Statistical significance: *$p < 0.05$.

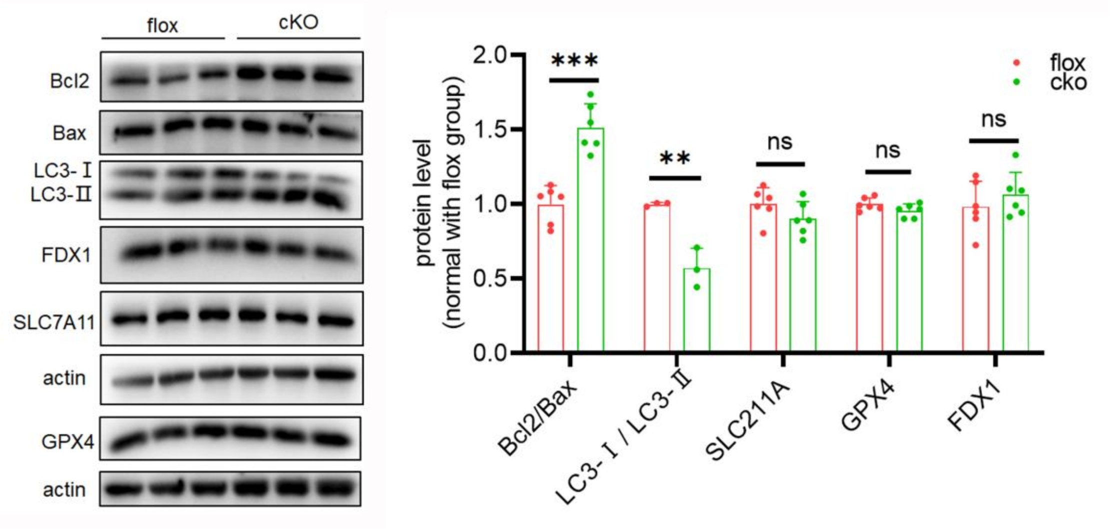

Figure S2. Microglial RhoA deficiency inhibits apoptosis, promotes autophagy, and does not influence ferroptosis or cuproptosis. (Bcl2 and Bax are the markers of apoptosis, LC3-I and LC3-II are the markers of autophagy, FDX1 and SLC7A11 are the markers of ferroptosis, GPX4 is the marker of cuproptosis.). Data are presented as mean ± SD; each dot represents an individual mouse. Statistical significance: ns means no significance; $**p < 0.01$; $***p < 0.001$.

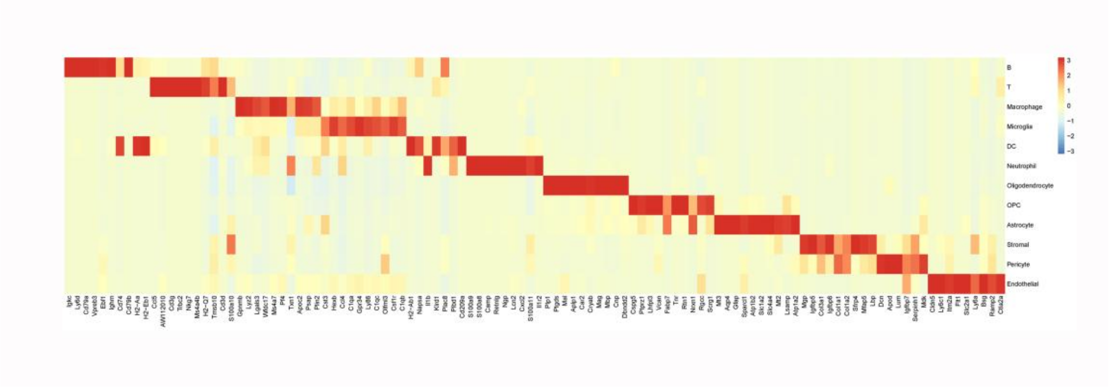

Figure S3 Heat map showing the top DEGs in each cluster of spinal cord's cells based on adjusted p values. The color depth represents the average expression level of the gene in the cluster. (B means B cell; T means T cell; DC means dendritic cell; OPC means Oligodendrocyte Precursor Cell)

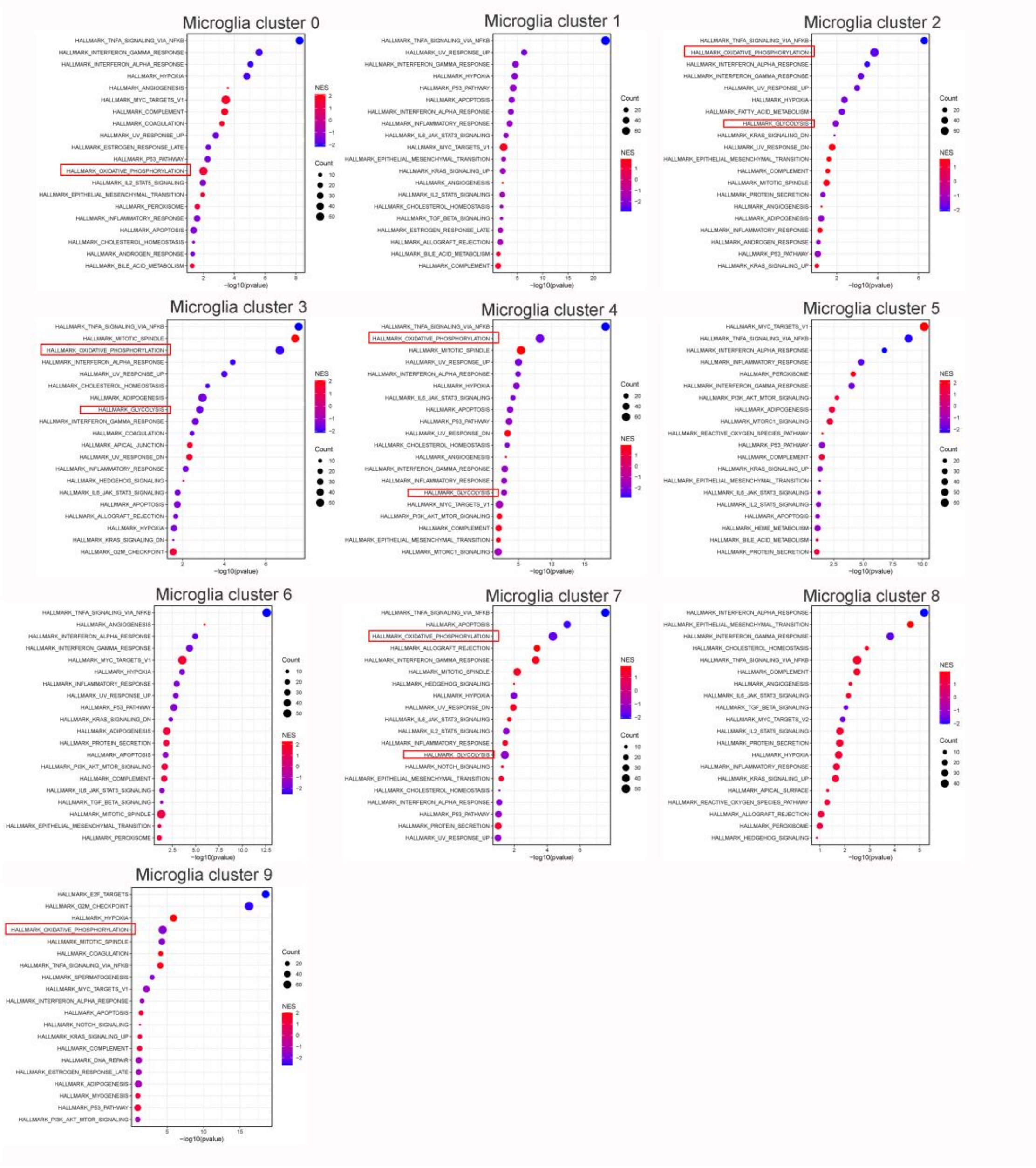

Figure S4 GSEA enriched for different microglial cluster. For each cluster, the GSEA results display the top 20 significantly enriched pathways, with oxidative phosphorylation and glycolysis prominently highlighted.

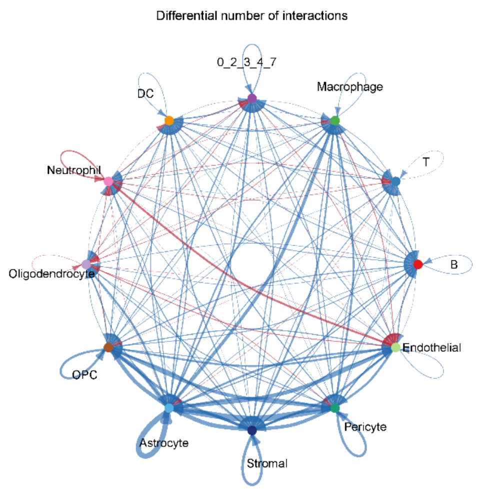

Figure S5. CellChat analysis reveals the connection between the microglia subcluster with significantly different oxidative phosphorylation processes and other cells. (0_2_3_4_7 means microglia subcluster 0, 2, 3, 4, 7; B means B cell; T means T cell; DC means dendritic cell; OPC means Oligodendrocyte Precursor Cell)

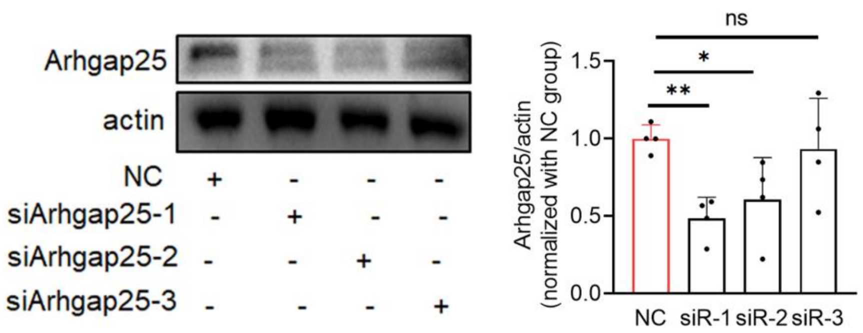

Figure S6. siRNA knocked down Arhgap25 in primary cultured microglia.

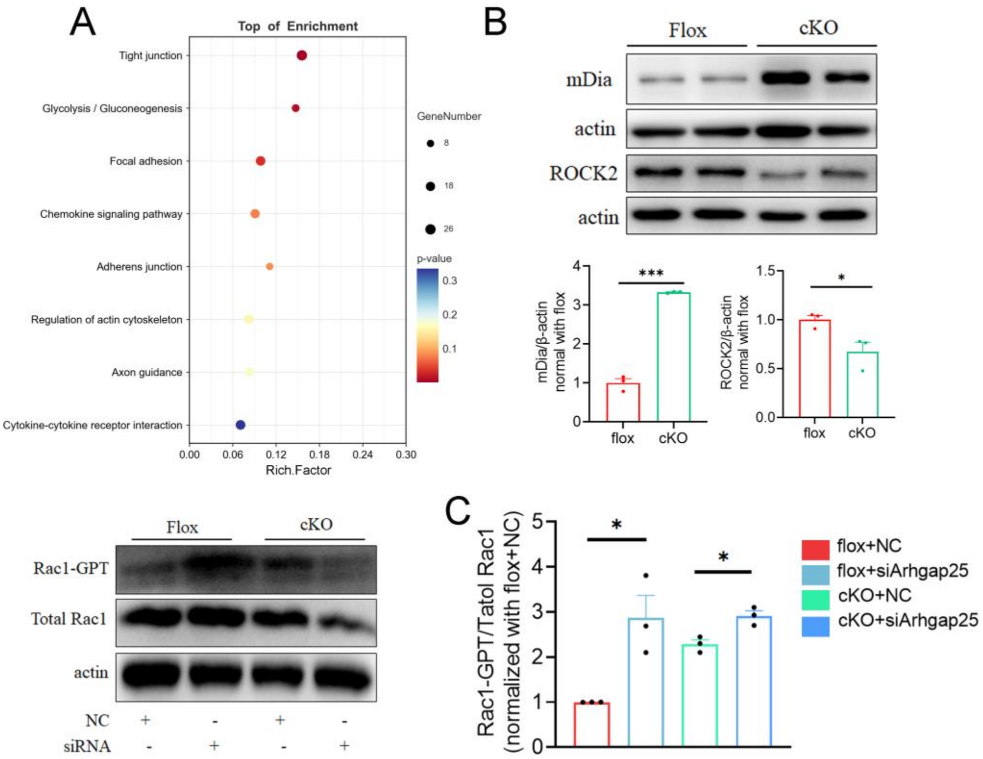

Figure S7. RhoA deletion upregulates mDia expression and enriches cytoskeleton-related pathways.

(A) KEGG enrichment analysis of differentially expressed genes after RhoA deletion in microglia. While glycolysis/gluconeogenesis pathways are ranked at the top, cytoskeleton-related pathways such as focal adhesion, adherens junction, and regulation of actin cytoskeleton are also enriched, though at a lower rank. (B) Western blot analysis and quantification of mDia and ROCK2 expression in primary microglia from flox and cKO mice. (C) Rac1 activation was assessed using a Rac1-GTP pull-down assay in microglia transfected with NC or Arhgap25 siRNA. Data are presented as mean ± SD; each dot represents an individual mouse. Statistical significance: $*p < 0.05$.

Table S1 si*Arhgap25* sequence information

Product number	Name	Target sequence
siG2112090126260944	si*Arhgap25*-1	CGACTACAAACTCCTGAAA
siG2112090126262036	si*Arhgap25*-2	ACACCAACCTCAGCAGTTA
siG2112090126263128	si*Arhgap25*-3	GCATCAAAGTGGAGCGAAA

Table S2 Microglia sequencing data from GEO database.

GEO accession	Library type	Description
GSE196928	RNA-seq	crush injury
GSE174549	RNA-seq	contusion injury
GSE249615	RNA-seq	contusion injury
GSE93249	RNA-seq	contusion injury

Table S3 Antibodies used in this study

Antibodies	Source species	Identifier	RRID	dilution	Company
**IBA-1**	Goat	NB100-1028	AB_3148646	1:500 (IF)	Novus Biologicals (Colorado, USA)
**IBA-1**	Rat	019-19741	AB_839504	1:500 (IF)<br>1:1000 (WB)	Wako (Kawasaki, Japan)
**GAP43**	Rabbit	ab75810	AB_1310252	1:400 (IF)	abcam (Cambridge，UK）
**NeuN**	Rabbit	ab177487	AB_2532109	1:400 (IF)	abcam (Cambridge，UK）
**RhoA**	Mouse	sc-418	AB_628218	1:400 (IF)<br>1:1000 (WB)	Santa cruz (California, USA)
**PCNA**	Mouse	60097-1-Ig	AB_2236728	1:400 (IF)<br>1:1000 (WB)	PROTEINTECH GROUP (Wuhan, China)
**PH3**	Rabbit	ET1601-30	AB_3069613	1:1000 (WB)	HUABIO (Hangzhou, China)
**Ki67**	Rabbit	ab15580	AB_443209	1:400 (IF)<br>1:1000 (WB)	abcam (Cambridge，UK）
**p-Chk1**	Rabbit	AP0578	AB_2770995	1:1000 (WB)	ABclonal (Wuhan, China)
**Chk1**	Rabbit	T55063	In application	1:1000 (WB)	Abmart (Shanghai, China)
**Cdk1**	Rabbit	T55061	In application	1:1000 (WB)	Abmart (Shanghai, China)
**Cdk2**	Rabbit	A0094	AB_2861449	1:1000 (WB)	ABclonal (Wuhan, China)
**β-Actin**	Rabbit	GB15003	AB_3083699	1:1000 (WB)	Servicebio (Wuhan, China)
**Tubulin**	Mouse	ab7291	AB_2241126	1:1000 (WB)	abcam (Cambridge，UK）
**Bcl2**	Rabbit	ab182858	AB_2715467	1:1000 (WB)	abcam (Cambridge，UK）
**Bax**	Rabbit	ab32503	AB_725631	1:1000 (WB)	abcam (Cambridge，UK）
**LC3B**	Rabbit	T55992	AB_2929010	1:1000 (WB)	Abmart (Shanghai, China)
**PDX1**	Rabbit	TD7170	In application	1:1000 (WB)	Abmart (Shanghai, China)
**SLC7A11**	Rabbit	TD12509	AB_2936892	1:1000 (WB)	Abmart (Shanghai, China)

**GPX4**	Rabbit	TD6701	In application	1:1000 (WB)	Abmart (Shanghai, China)
**MBP**	Mouse	sc-66064	AB_832661	1:400 (IF)<br>1:1000 (WB)	Santa cruz (California, USA)
**CD68**	Rabbit	ab283654	AB_2922954	1:400 (IF)<br>1:1000 (WB)	abcam (Cambridge, UK）
**Arhgap25**	Rabbit	A13664	AB_2760525	1:400 (IF)<br>1:1000 (WB)	ABclonal (Wuhan, China)
**HIF-1α**	Rabbit	A22041	In application	1:400 (IF)<br>1:1000 (WB)	ABclonal (Wuhan, China)
**HIF1AN**	Rabbit	YP-Ab-12574	In application	1:1000 (WB)	UpingBio technology (Hangzhou, China)
**PKM2**	Rabbit	T55764	In application	1:1000 (WB)	Abmart (Shanghai, China)
**LDHA**	Rabbit	T58276	In application	1:1000 (WB)	Abmart (Shanghai, China)
**HK2**	Rabbit	HA500186	AB_3071283	1:1000 (WB)	HUABIO (Hangzhou, China)
**Alexa 568-conjugated Goat anti-Mouse**	Mouse	A-11004	AB_2534072	1:800 (IF)	Invitrogen (MA, USA)
**Alexa 568-conjugated Goat anti- Rabbit**	Rabbit	A-11008	AB_143165	1:800 (IF)	Invitrogen (MA, USA)
**Alexa 568-conjugated Donkey anti-Goat**	Goat	A-11057	AB_2534104	1:800 (IF)	Invitrogen (MA, USA)
**Alexa 488-conjugated Goat anti- Mouse**	Mouse	A-11001	AB_2534069	1:800 (IF)	Invitrogen (MA, USA)
**Alexa 488-conjugated Goat anti-Rabbit**	Rabbit	A-11008	AB_143165	1:800 (IF)	Invitrogen (MA, USA)
**HRP-conjugated Goat anti-Mouse IgG**	Mouse	AS003	AB_2769851	1:6000 (WB)	ABclonal (Wuhan, China)
**HRP-conjugated Goat anti-Rabbit IgG**	Rabbit	AS014	AB_2769854	1:6000 (WB)	ABclonal (Wuhan, China)

Note: IF=Immunofluorescent staining, WB= Western blotting
