# Supplementary material for: Loss of RhoA in microglia disables glycolytic adaptation and impairs spinal cord injury recovery through Arhgap25/HIF-1α pathway
Source: Cell Death Dis. 2025 Aug 22;16(1):636. doi: 10.1038/s41419-025-07947-9 (PMC12373832; doi:10.1038/s41419-025-07947-9)

WB Raw data in

Loss of RhoA in microglia disables glycolytic adaptation and impairs spinal cord injury recovery through Arhgap25 /HIF-1α pathway

Jiale Cai [a, b†], Xinya Zheng [a†], Xiongbo Luo [a], Wenli Cui [a], Xinrui Ma [a, b], Shuyi Xu [a, b], Lanya Fu [a], Jiaqi Zhang [a], Yizhou Xu [a, c], Yunlun Li [a], Ye He [a], Xianghai Wang [a, b], Jiasong Guo [a, b, c*]

a. Department of Histology and Embryology, Guangdong Provincial Key Laboratory of Construction and Detection in Tissue Engineering, National Demonstration Center for Experimental Education, School of Basic Medical Sciences; Department of Neurosurgery, Institute of Brain Diseases, Nanfang Hospital; Southern Medical University, Guangzhou, Guangdong Province, China

b. Key Laboratory of Mental Health of the Ministry of Education, Guangdong-Hong Kong-Macao Greater Bay Area Center for Brain Science and Brain-Inspired Intelligence, Guangdong Province Key Laboratory of Psychiatric Disorders, Guangzhou, Guangdong Province, China

c. Department of Spine Orthopedics, Zhujiang Hospital, Southern Medical University, Guangzhou, Guangdong Province, China

† Jiale Cai and Xinya Zheng contributed equally to this work.

*Correspondence to

Professor Jiasong Guo, Southern Medical University, Guangzhou 510515, China; jiasongguo@smu.edu.cn.

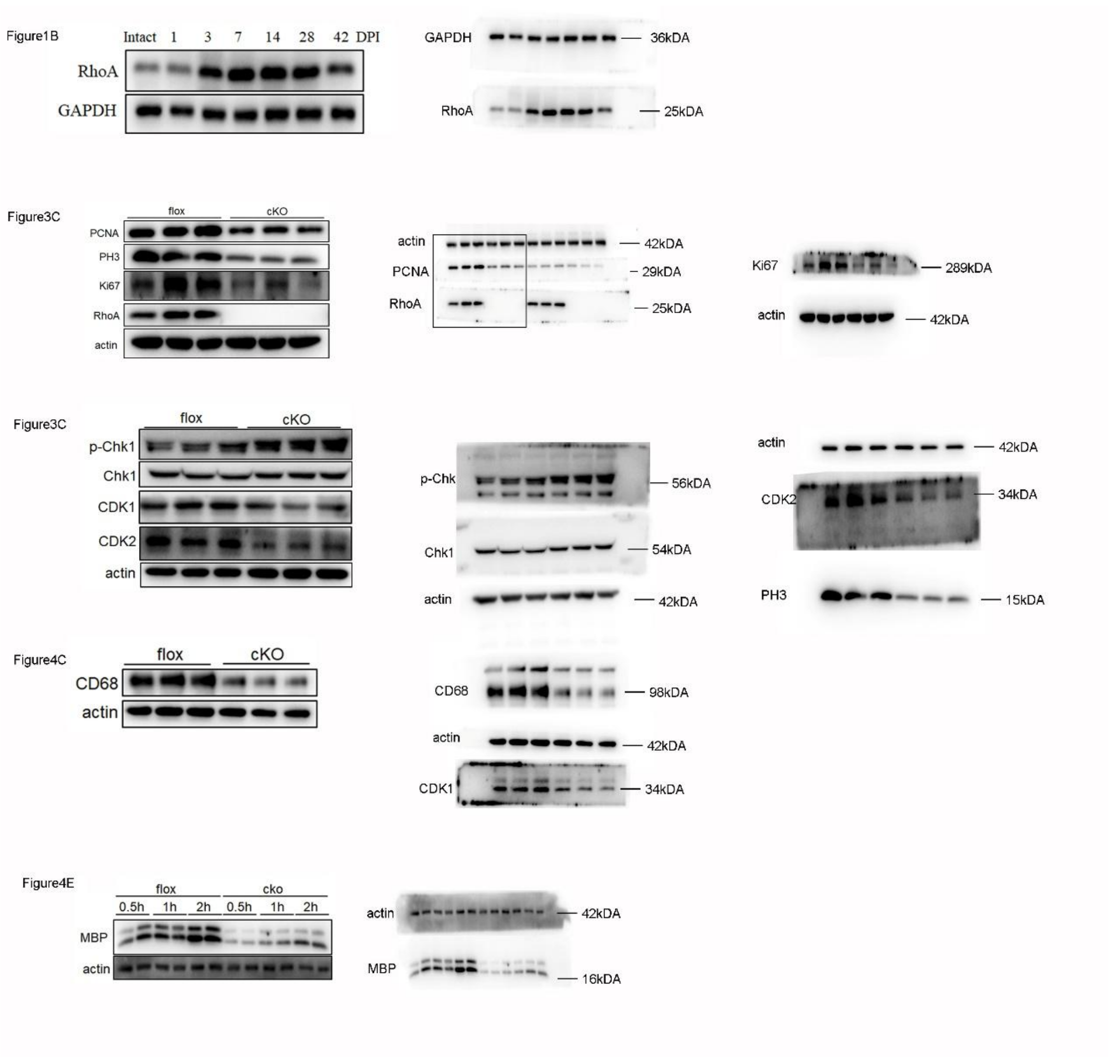
Figure1B
Intact 1 3 7 14 28 42 DPI
RhoA
GAPDH
GAPDH — 36kDA
RhoA — 25kDA
Figure3C
flox cKO
PCNA
PH3
Ki67
RhoA
actin
actin — 42kDA
PCNA — 29kDA
RhoA — 25kDA
Ki67 — 289kDA
actin — 42kDA
Figure3C
flox cKO
p-Chk1
Chk1
CDK1
CDK2
actin
p-Chk — 56kDA
Chk1 — 54kDA
actin — 42kDA
actin — 42kDA
CDK2 — 34kDA
PH3 — 15kDA
Figure4C
flox cKO
CD68
actin
CD68 — 98kDA
actin — 42kDA
CDK1 — 34kDA
Figure4E
flox cko
0.5h 1h 2h 0.5h 1h 2h
MBP
actin
actin — 42kDA
MBP — 16kDA

Figure 6D

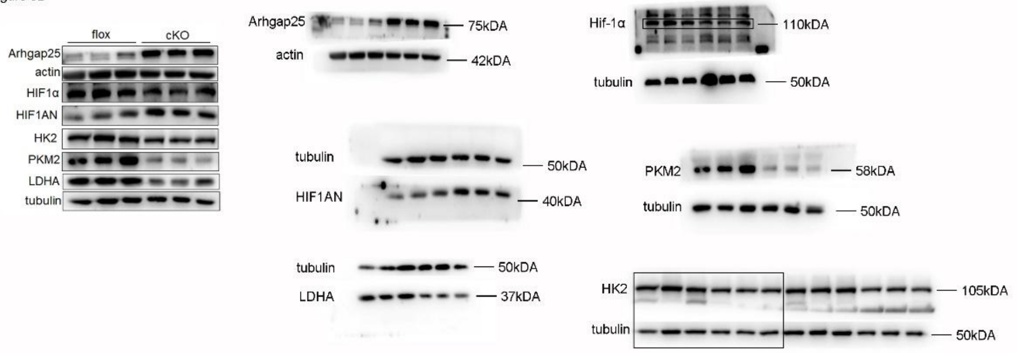

Figure 7A

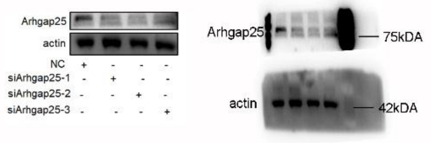

Figure 7B

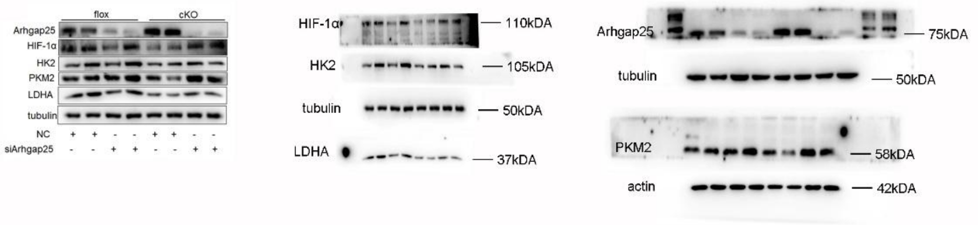

Figure 7C

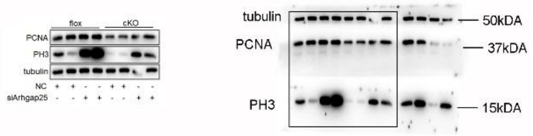

Figure 8C

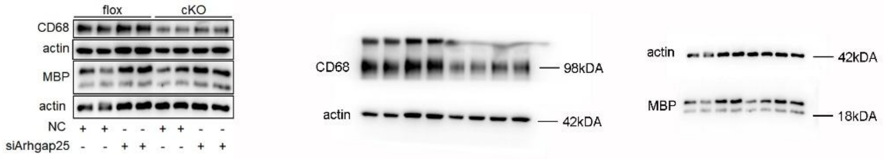

Supplement: Supplementary file 2 — WB-RAW-DATA [file 41419_2025_7947_MOESM2_ESM.pdf]
